# Qualitative and Quantitative Changes in Total Lipid Concentration and Lipid Fractions in Liver Tissue of Periparturient German Holstein Dairy Cows of Two Age Groups

**DOI:** 10.3389/fvets.2022.814808

**Published:** 2022-03-15

**Authors:** Kirsten B. Theinert, Teja Snedec, Fabian Pietsch, Sabrina Theile, Anne-Sophie Leonhardt, Joachim Spilke, Stefan Pichelmann, Erik Bannert, Kristin Reichelt, Gabriele Dobeleit, Herbert Fuhrmann, Walter Baumgartner, Melanie Schären-Bannert, Alexander Starke

**Affiliations:** ^1^Clinic for Ruminants and Swine, Faculty of Veterinary Medicine, University of Leipzig, Leipzig, Germany; ^2^Biometrics and Informatics in Agriculture Group, Institute of Agriculture and Nutrition, Martin-Luther-University, Halle, Germany; ^3^Department of Psychology, University of Fribourg, Fribourg, Switzerland; ^4^Institute of Physiological Chemistry, Faculty of Veterinary Medicine, University of Leipzig, Leipzig, Germany; ^5^University Clinic for Ruminants, University of Veterinary Medicine, Vienna, Austria

**Keywords:** triacylglycerol, phospholipid, fatty liver, free fatty acid, cholesterol ester

## Abstract

Fatty liver syndrome (FLS) is a common disease in high-producing dairy cows. Studies in humans suggest that the different hepatic lipid fractions play a role in this context. In dairy cows, little is known about the composition of fat stored in the liver, its periparturient dynamics, and the effect of cows' age. Therefore, our goal was to generate primary data in healthy cows to serve as reference values for future studies. Eight healthy German Holstein cows (2nd lactation, *n* = 3; ≥3rd lactation, *n* = 5) were examined 14 d antepartum and 7, 28, and 42 d postpartum. The examinations included clinical assessment, liver biopsy, blood sampling, and recording of milk yield. Total lipids (TL) in liver tissue were measured gravimetrically. The TL were separated into lipid fractions (triacylglycerol, TAG; phospholipids, PL; non-esterified fatty acids, NEFA; and cholesterol esters) using thin-layer chromatography, followed by gas chromatography for fatty acid determination. Concentrations of NEFA, ß-hydroxybutyrate, and cholesterol were analyzed in blood. Concentrations of TL, TAG, NEFA, and cholesterol esters in liver tissue and NEFA in blood increased in the periparturient period. The older cows had higher hepatic TL, TAG, and PL concentrations, higher relative hepatic concentrations of TAG in TL, higher NEFA concentrations in blood, a greater decrease in body condition, and higher milk yields between d 9 and 40 than the younger cows. We proposed that due to higher milk yield, older cows mobilized and deposited more fat in the liver, and the increase in hepatic TAG concentration was longer-lasting than in younger cows. Higher levels of structural lipids (PL) in older cows could be explained by higher demand for storage of TAG and cholesterol esters in lipid droplets or for the export of TAG via very-low-density lipoproteins. Results show that hepatic fat storage is a reversible process and does not necessarily cause clinical disease. Nevertheless, older cows have a more sustained and greater increase in hepatic TAG concentration, which may explain their increased risk of FLS. The results are limited in their extrapolation due to the small sample size and thereby possible selection bias but present a valuable basis for future studies.

## Introduction

Fatty liver syndrome (FLS) in dairy cows is associated with metabolic and infectious diseases, reproductive problems, displaced abomasum, and lameness (1–3). In addition, cows with fatty liver are predisposed to liver insufficiency (4, 5). The economic impact of FLS on the dairy industry is significant because of production losses, treatment costs, and premature culling of affected cows (4). Parity has an effect on hepatic fat content as multiparous cows have a higher hepatic total lipid (TL) and triacylglycerol (TAG) content after calving than primiparous cows (6). Multiparous cows have an increased risk of FLS (6–9) and are more susceptible to periparturient diseases (4, 10, 11) and downer cow syndrome (9). The age of the cow seems to play an important role. Cows with severe fatty liver were considerably older (mean age 6.2 y) than reference cows (mean age 5.4 y) or cows with mild fatty liver [mean age 4.4 y; (9)]. Some cows with severe fatty liver survive, some die despite treatment (1, 9). The reasons for this are not yet known. The cause of death was considered to be liver failure based on the absence of other diseases (9). Studies in humans have shown that fat accumulation in the liver alone is not a cause of liver failure (12). In human medicine, the cause of steatohepatitis is now thought to be the lipotoxic effect of certain lipid fractions, NEFA, and fatty acid metabolites (12, 13).

Earlier studies in dairy cows point toward a role of these lipid fractions in the pathogenesis of FLS: the increase in TL content in the periparturient period is essentially caused by an increase in TAG (14). Starke et al. (15) showed in a trial involving 150 German Holstein dairy cows (in a single sampling of liver tissue during routine right flank omentopexy for correction of left displaced abomasum) that the hepatic TAG content increased linearly to a breakpoint of ~100 mg/g of liver fresh weight (FW; breakpoint 107 mg/g FW), at which point it reached a plateau. It is possible that with a TL content >100 mg/g FW, the absolute and relative concentrations of other lipid fractions including non-esterified fatty acids (NEFA), cholesterol, or cholesterol esters (CE), or both, increase (15). In contrast, a study involving 13 slaughtered dairy cows with high serum levels of free fatty acids showed that the proportion of CE is lower in fatty liver than in healthy bovine liver (16). However, data illustrating the timely dynamics in this context are lacking. Cholesterol is required for the export of TAG as very-low-density lipoproteins [VLDL; (17)]. The liver responds to the increased demand for cholesterol at the start of lactation with the upregulation of key enzymes for hepatic cholesterol biosynthesis (17). However, the plasma concentration of VLDL remains low, and the reasons for this are not clear (17). Phospholipids (PL) may be the limiting factor for VLDL secretion (17) because studies have shown that the hepatic content of PL did not change significantly in the periparturient period (18).

Many studies have relied on blood analysis to describe the alterations in liver lipid metabolism and the changes in circulating lipid fractions. Numerous studies described a positive association between the concentration of NEFA in blood and the fatty liver content of periparturient dairy cows [e.g., as reviewed by (19, 20)]. The peripartum dynamics and changes relating to lipomobilization and FLS were also described for TAG (21–24), PL (18, 24–27), and CE (17, 24, 28). Blood analysis is a feasible method for routine evaluation, but the profiles generated are the result of different metabolic processes in various organs and tissues. For evaluation and investigation of liver metabolism liver biopsy samples were shown to provide better insight into liver metabolism [discussed in a recent publication by Schären et al. (29) comparing metabolomic profiles in blood and liver samples]. This was supported by a recent study by Fry et al. (30) in which cytology had a higher correlation for assessing hepatic lipid content and liver TAG than plasma levels of NEFA and BHB.

To investigate the potential lipotoxic effects of hepatic lipid fractions in the pathogenesis of FLS, it is therefore essential to characterize the dynamics of these lipid fractions in the liver itself during the transition period (with multiple samples across time) in healthy cows. The data generated would serve as future reference values because similar studies are lacking (pubmed research “fatty acid composition, liver, multiparous, cow” November 4, 2021). However, the methods used, including TL extraction, thin-layer chromatography, and gas chromatography are labor-intensive and expensive. In addition, a high level of expertise, particularly for *in-vivo* collection of large (>500 mg) liver biopsy samples, is required. Based on the results of earlier studies, a sampling frequency of at least four separate sampling time points in the periparturient period appeared sensible for our study (31). Our experimental design represents a compromise between frequency of sampling and number of cows examined.

The aim of this study was to generate primary data in regard to alterations in hepatic lipid fractions throughout the periparturient period, allowing to identify possible timely dynamics and interrelations, and to develop a more targeted approach in future studies involving diseased cows.

The risk of FLS and associated periparturient diseases increases with age in dairy cows. We therefore hypothesized that the hepatic TL content and the absolute and relative amounts of the lipid fractions in the liver differ between younger (2nd) and older (3rd or greater lactation) German Holstein dairy cows in early lactation. Primiparous cows were not included in the study because they are not mature at calving (32) and therefore have a fundamentally different metabolic state compared with multiparous cows (33).

## Materials and Methods

### Research Permit

This study was part of a larger project focusing on metabolic disorders of dairy cows during the transition period. The experiment was conducted in accordance with the German Animal Welfare Act and the Saxony Veterinary and Food Safety Office Landesdirektion Sachsen, permit number TVV 33/15. Individual results from the associated overall project have already been published elsewhere (29, 34–36).

### Cows

The study was conducted in a commercial dairy farm with 660 German Holstein cows in Saxony. The rolling average milk production was 10,747 kg with 3.73% fat and 3.33% protein, and cows were housed in a free-stall and fed a total mixed ration (TMR). A detailed description of the study setup and husbandry routines (e.g., feeding, grouping) are described by Schären et al. (29). Briefly, for the overall trial 87 cows were selected from the herd. Inclusion criteria at 14 d antepartum (calculated from the expected calving date) were: ≥2nd lactation, pregnant, clinically healthy, no treatment 28 d prior to first sampling or persisting withdrawal period (exception: dry-off treatment), and expected dry period longer than 70 d. Seven animals were excluded during the trial for the following reasons: downer cow syndrome before calving, fetal mummification, death attributable to uterine prolapse, severe leg injury, fractious cow, misidentification (no correspondence between ear-tag and collar-number at some point during the trial), and incorrect pen allocation (29). The final number of cows included in the study was 80. There were two treatment groups consisting of *n* = 20 cows each and one placebo group (*n* = 40). The two treatment groups received Catosal® at the recommended dose of 5 ml (as registered in Germany) or twice the recommended dose of 10 ml/100 kg body weight (**BW**), 10% butaphosphan, and 0.005% cyanocobalamin. The placebo-group received 5 ml or 10 ml NaCl 0.9%/100 kg BW. The cows were assigned randomly to the groups using a lottery system, and the treatment was triple blinded. The treatments were administered intravenously at six time points: 7, 6, and 5 d a.p. and 1, 2, and 3 d postpartum. For this trial, eight clinically healthy cows in their 2nd to 6th lactation were recruited from the placebo group of the overall study. Of the 40 cows in the placebo group, 22 were considered healthy throughout the study period based on the criteria described in the next section. Of these 22 cows, three of six 2nd lactation animals and five of 16 ≥3rd lactation animals were chosen randomly. Lactation 2 cows (Lact2): age, 3.5 y [3.4–3.7 y, mean (min-max)]; BCS 14 d a.p., 3.0 [2.8–3.5; according to Edmonson et al. (37)]; height at withers, 142 cm (139–146 cm); BW, 738 kg [712–766 kg; weighed using a Texas Trading Squeeze Chute (Version S 04 with weighing scale)]; and 305-day milk yield in previous lactation, 9,749 kg (8,865–10,436 kg). For lactation 3 or greater cows (Lact≥3): age, 5.2 y (4.0–7.5 y); number of lactations, 3.8 (3.0–6.0); BCS 14 d a.p., 3.0 (2.5–3.3); height at withers, 148 cm (143-152 cm); BW, 757 kg (712–794 kg); 305-day milk yield in previous lactation, 11,861 kg (10,147–14,470 kg).

### Clinical and Laboratory Monitoring

The cows were monitored clinically from 14 d a.p. until the end of the study period 42 d p.p. by trained veterinarians who stayed on-site throughout the trial. They were supervised by a diplomate of the European College of Bovine Health Management and/or a specialized buiatrician. The clinical status of the cows was recorded throughout the trial using a small and large standardized protocol for daily and weekly physical examinations, respectively. Scoring was based on the clinical examination described by Rosenberger et al. (38) and included the evaluation of rectal temperature, heart and respiratory rates, rumen fill and stratification, abdominal wall tension, lameness, skin, jugular veins, hygiene score, and feces, auscultation of the lungs and heart for abnormal sounds as well as the rumen for the frequency and intensity of contractions, auscultatory percussion of the left and right abdominal walls, and transrectal palpation. The udder and milk were assessed by well-trained employees of the farm (29). The clinical findings were transferred to a clinical scoring system for different health traits as described by Schären et al. (29). A cow was considered healthy when no abnormal clinical findings were noted throughout the study period and no treatments were administered.

Blood samples were collected from a jugular vein into plastic tubes without anticoagulant for the determination of the NEFA concentration in serum, and into plastic tubes containing lithium heparin for the measurement of plasma concentrations of albumin, ß-hydroxybutyrate (BHB), bilirubin, creatinine, cholesterol, glucose, lactate, urea, triacyglycerol, calcium, chloride, potassium, magnesium, sodium, phosphorus, and total protein, and the activities of alkaline phosphatase, alanine aminotransferase, aspartate aminotransferase, creatine kinase, gamma glutamyltransferase, and glutamate dehydrogenase. The chloride concentration of voided urine was determined. The analyses of blood and urine samples were done in the laboratory of Large Animal Clinics, Faculty of Veterinary Medicine, University of Leipzig using a Cobas c 311 system (Roche Diagnostics, Mannheim, Germany). A more detailed description was provided by Schären et al. (29). All laboratory results were within the reference intervals (39).

### Liver Biopsy

Liver biopsy (1–2 g liver FW) was done at 14 d a.p. and 7, 28, and 42 d p.p. under local anesthesia [Isocain ad. us. vet. 20 mg/ml; Selectavet, 20 mg/ml procaine hydrochloride (corresponding to 17.3 mg/ml procaine and 0.025 mg/ml epinephrine, Weyarn/Holzolling, Germany)] using the technique described by Gohlke et al. (40). The biopsy samples were stored for about 1 week in liquid nitrogen and then at −80°C until processing. Difficulty in predicting parturition, variable farm management schedules, and high operator workload resulted in minor deviations in the actual from the scheduled sampling days: 14 [11, 13–6; scheduled sampling day (actual sampling day: mean, earliest-latest)] d a.p.; 7 (8, 6–9) d p.p., 28 d (28, 26–29) d p.p., and 42 (42, 40–44) d p.p. The deviations from scheduled sampling days were taken into account in the statistical analysis.

### Determination of Total Lipid Content and Lipid Fractions

The TL content, the lipid fractions, and the fatty acid composition of each lipid fraction in the biopsy samples were determined as described by Adolph et al. (41). The TL content (mg/g liver FW) was measured gravimetrically, and the lipid fractions were separated using thin-layer chromatography. Gas chromatography was used to analyze the fatty acid composition of each lipid fraction (μmol/g liver FW). The concentrations of the individual fatty acids within a lipid fraction were then used to calculate the total concentrations of TAG, PL, NEFA, and CE (converted to mg/g).

### Presentation of the Results

The results of the analyses of the TL content and lipid fractions of liver tissue are presented as concentrations (mg/g liver FW) to provide an overview of the magnitude of fatty infiltration of the liver. The lipid fractions are also given as percentages of hepatic TL content to illustrate the changes in liver fat composition in the periparturient period. The plasma concentrations of BHB and cholesterol (mmol/l) and serum concentrations of NEFA (μmol/l), the BCS scores, mean BW, and milk yields reflect periparturient metabolic changes.

### Statistical Analysis

#### Total Lipid Content, Lipid Fractions and Their Percentages of the TL Content, Concentrations of NEFA, BHB, and Cholesterol in Blood, and BCS

Group (Lact2 and Lact≥3) and sampling days and interactions between these variables were fixed qualitative effects. Repeated measures within cows were taken into account by choosing an appropriate covariance structure [ar(1), toep(2), cs, un, vc] with the help of the Akaike information criterion with correction for finite samples (AICC). Because the scheduled sampling times could not be followed in all cases, deviations from the scheduled sampling days were considered an additional independent variable via a linear covariate. The covariable was included in the evaluation model if the associated regression coefficients were significant, which occurred in 23.1% of the cases. Some data were transformed to obtain normal distribution of the residuals. For absolute concentrations, log(e) and square root transformations were tested, and for percentages of lipid fractions, arcsine square root transformation and log(e) transformations were tested. Type of transformation was chosen based on the best fit to the normal distribution according to the Shapiro-Wilk test. For traits that required transformation, the least square means (LSM) given in our results represent back transformations.

#### Milk Yield

The effect of days in milk (DIM) on milk yield was described as a 2nd order fractional:


(1) $$\begin{array}{r} f\left(t\right)=\beta_{0}+\beta_{1}\times x_{1}+\beta_{2}\times x_{2} \end{array}$$

*where*


(2) $$\begin{array}{r} x_{1}=\log\left(t\right),\;x_{2}=t^{1}\;and\;t=\;\frac{DIM}{42} \end{array}$$

The regression coefficients were considered specific to the groups. This approach was obtained by systematic search for p1, p2 in the interval (−2; 2) when comparing the variants with AICC. Repeated measures within cows were modeled using the Toeplitz(10) function.

#### BW

Since no normal distribution of the residuals could be secured for BW assuming a linear mixed model (see above), a rank analysis according to Brunner and Munzel was required for the evaluation of this trait (42).

#### General Remarks

With the exception of BW, for which a model based on ranks was used, a linear mixed model was used for all other variables because they involved repeated measures. Degree of freedom approximation based on Kenward and Roger (43) was required for the F-test and the analysis of differences and confidence intervals of the LSM in the original or transformation scales. Differences between LSM were analyzed using a *t*-test and the Tukey-test. For the variables TL, absolute and relative amounts of the lipid fractions, NEFA, BHB and cholesterol in blood, and BCS, only comparisons with biological importance were analyzed. Therefore, the possible comparisons of groups and sampling days (28 comparisons in total) were limited to comparisons of sampling days within groups (6 comparisons each) and to comparisons of groups within sampling days (one comparison in total). Accordingly, F- and Tukey-tests of interactions are given only for these comparisons. All analyses were done using SAS 9.4 (SAS Institute 2016, Cary, NC, USA; proc univariate, proc mixed, proc iml).

## Results

**Figure 1:**
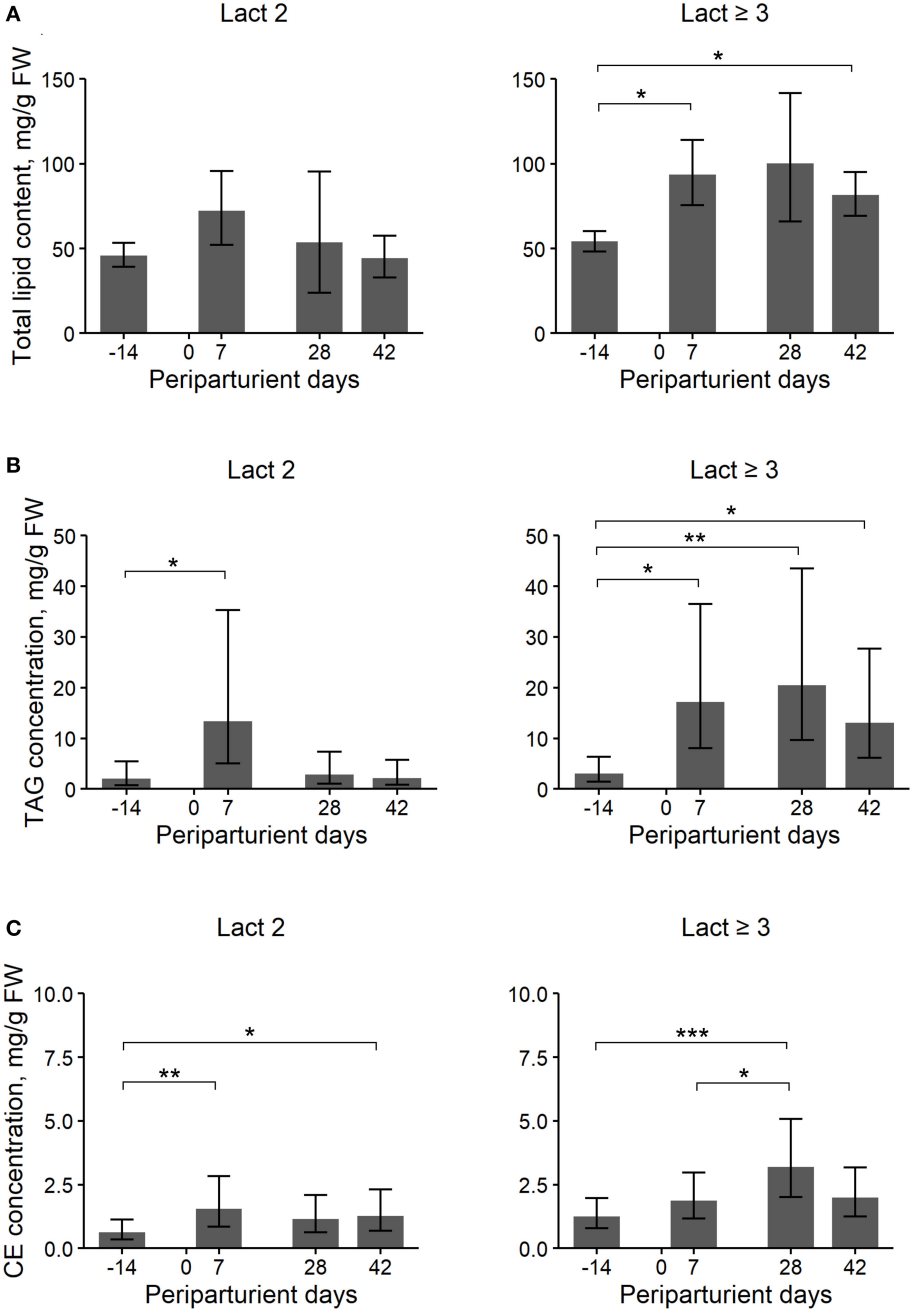
Least Square Means (LSM) and their 95% confidence intervals (error bars) for the hepatic total lipid (TL) **(A)**, triacylglycerol (TAG) **(B)**, and cholesterol ester (CE) **(C)** content (mg/g liver fresh weight, FW) at four periparturient sampling days [−14 = d 14 antepartum (a.p.); 0 = parturition; 7, 28, 42 = d 7, d 28, d 42 postpartum (p.p.)] for two groups (Lact2 = lactation 2, *n* = 3 and Lact≥3 = lactation 3 or greater, *n* = 5) of German Holstein cows. Significances between sampling days within groups (*P*-values F-test): TL: Lact2: *P* = *0.24*, Lact≥3: *P* = *0.03*; TAG: Lact2: *P* = *0.03*, Lact≥3: *P* < *0.01*; CE: Lact2: *P* < *0.01*, Lact≥3: *P* < *0.01*. Significant differences between days within group are marked with asterisks (*P < *0.05*; **P < *0.01*; ***P < *0.001*).

**Figure 2:**
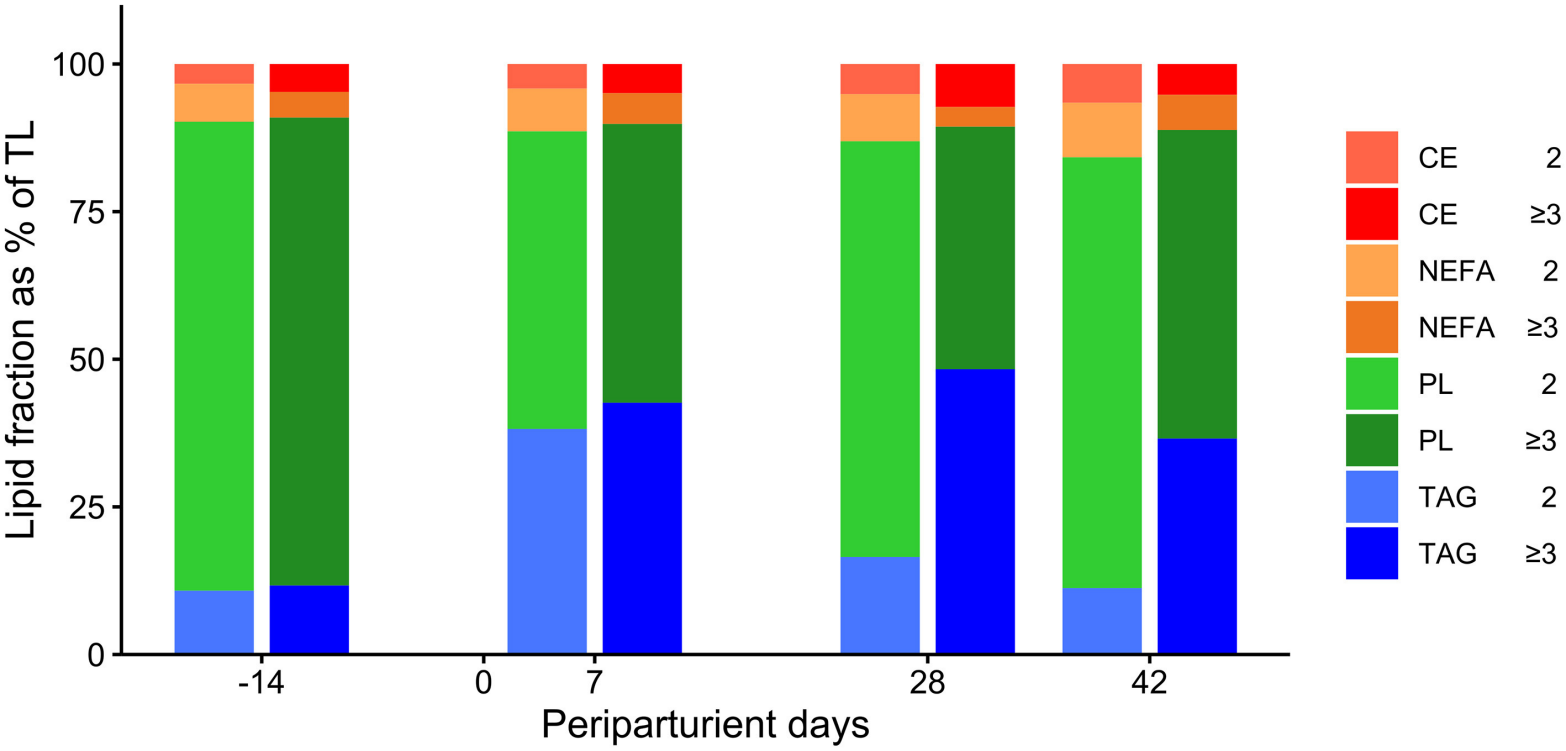
Percentage distribution of triacylglycerol (TAG), phospholipids (PL), non-esterified fatty acids (NEFA), and cholesterol esters (CE) of the total lipid content in liver tissue in German Holstein cows of groups Lact2 (lactation 2, *n* = 3) and Lact≥3 (lactation 3 or greater, *n* = 5) in the transition period [−14 = d 14 antepartum (a.p.); 0 = parturition; 7, 28, 42 = d 7, d 28, d 42 postpartum (p.p.)]. Significances between sampling days within groups (*P*-values F-test): TAG: Lact2: *P* = *0.07*, Lact≥3: *P* = *0.21*; PL: Lact2: *P* = *0.04*, Lact≥3: *P* < *0.01*; NEFA: Lact2: *P* = *0.48*, Lact≥3: *P* = *0.10*; CE: Lact2: *P* = *0.11*, Lact≥3: *P* = *0.14*.

**Figure 3:**
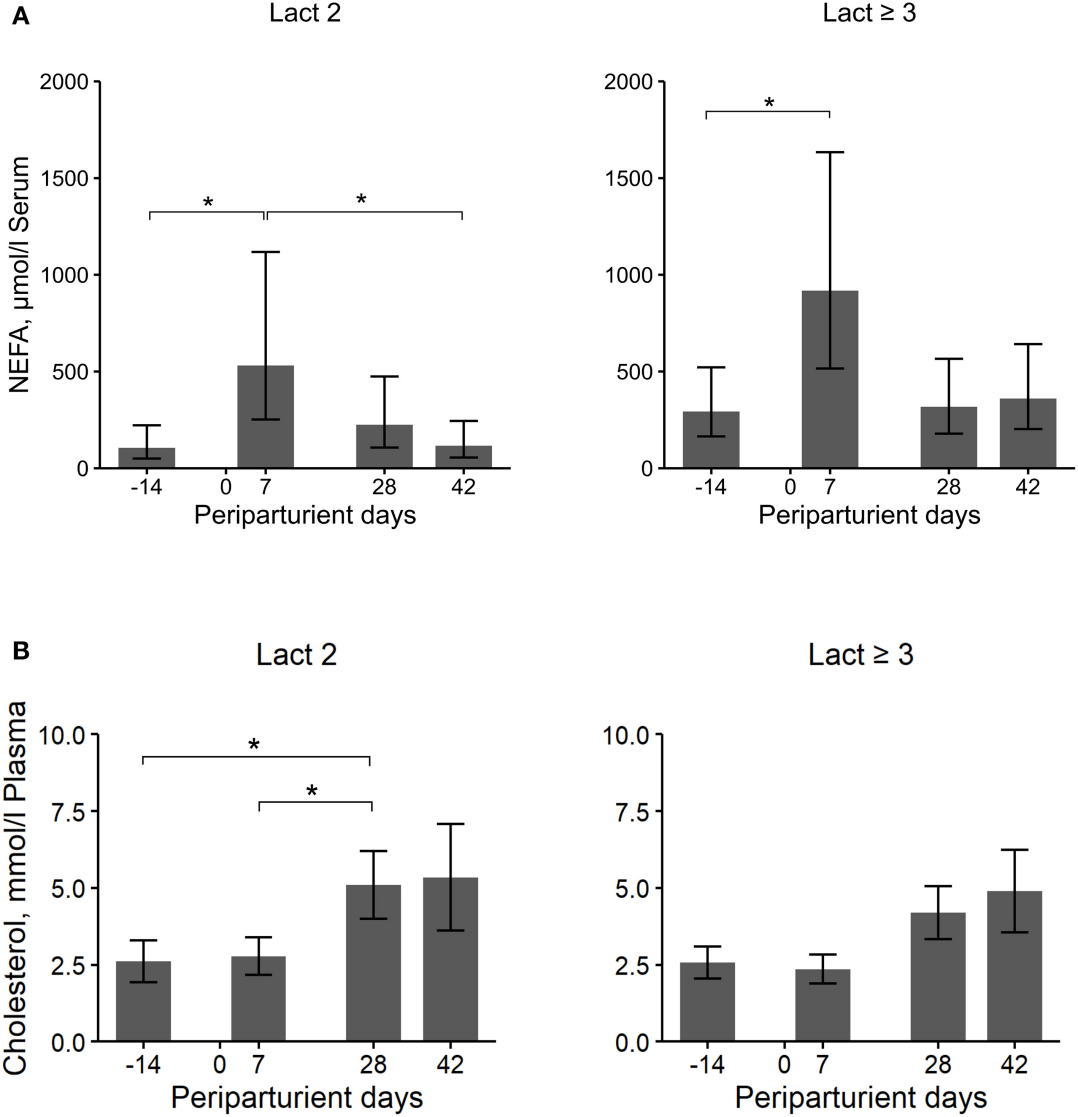
Least square means (LSM) and 95% confidence intervals (error bars) for the NEFA serum **(A)** and cholesterol plasma **(B)** concentration at four periparturient sampling days [−14 = d 14 antepartum (a.p.); 0 = parturition; 7, 28, 42 = d 7, d 28, d 42 postpartum (p.p.)] in two groups (Lact2 = lactation 2. *n* = 3; Lact≥3 = lactation 3 or greater, *n* = 5) of German Holstein cows. Significances between sampling days within groups (*P*-values F-test): NEFA serum: Lact2: *P* = *0.02*, Lact≥3: *P* = *0.04*; cholesterol plasma: Lact2: *P* = *0.04*, Lact≥3: *P* = *0.05*. Significant changes over time are marked with asterisks (*P < *0.05*).

**Figure 4:**
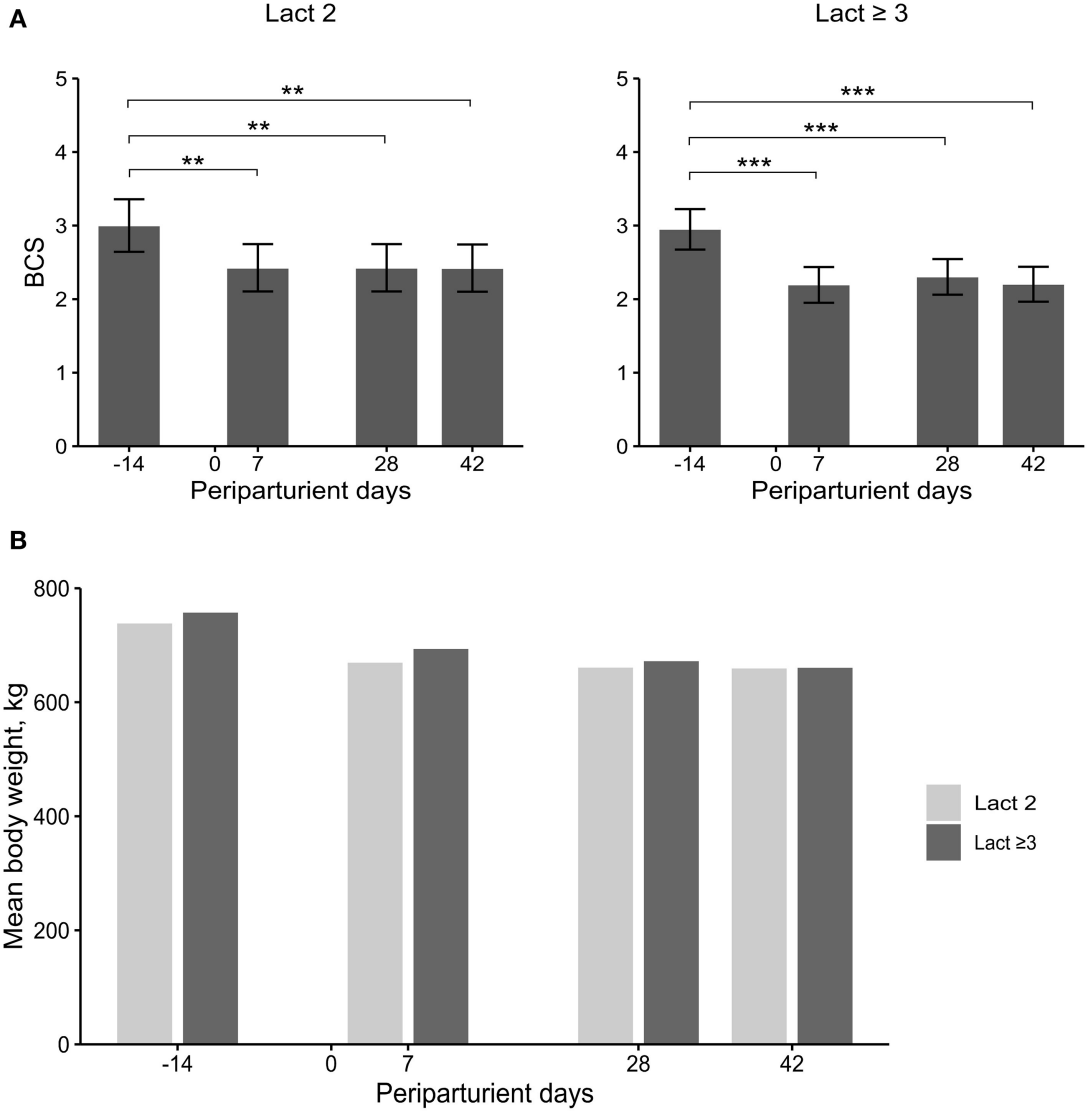
Least square means (LSM) and 95% confidence intervals (error bars) for the body condition score (BCS) **(A)** and mean body weight **(B)** at four periparturient sampling days [−14 = d 14 antepartum (a.p.); 0 = parturition; 7, 28, 42 = d 7, d 28, d 42 postpartum (p.p.)] in two groups (Lact2 = lactation 2, *n* = 3; Lact≥3 = lactation 3 or greater, *n* = 5) of German Holstein cows. Significances between sampling days within groups (*P*-values F-test): BCS: Lact2: *P* < *0.01*, Lact≥3: *P* < *0.01*; mean BW: d 14 a.p.: (*P* = *0.42*), d 7 p.p.: (*P* = *0.32*), d 28 p.p.: *P* = *0.50*, d 42 p.p.: (*P* = *0.78*). Significant changes over time are marked with asterisks (**P < *0.01*, ***P < *0.001*).

The comparisons of the main effects of groups and sampling days are provided in Tables 1, 2. Comparisons of the different sampling days within the respective groups are given in Figures 1–**4** for a selection of traits. The significant differences between the two groups within sampling days are provided in the text.

**Table 1:** Least square means ± standard errors and F-test results (*P-values*) for the main effects group and sampling day of total lipid (TL) content and the concentrations of lipid fractions triacylglycerol (TAG), phospholipids (PL), non-esterified fatty acids (NEFA), and cholesterol esters (CE; in mg/g liver fresh weight) as well as for the percent distributions of the lipid fractions TAG, PL, NEFA, and CE of TL.

	**Group** ^**1**^			**Sampling day** ^**2**^				
	**Lact2**	**Lact≥3**	***P-*value**	**14 d a.p**.	**7 d p.p**.	**28 d p.p**.	**42 d p.p**.	***P*-value**
**Concentrations**								
TL	54.5 ± 6.1^b^	82.6 ± 5.9^a^	0.02	50.9 ± 2.2^b^	83.9 ± 6.1^a^	76.3 ±11.1^ab^	62.7 ± 3.8^ab^	0.05
TAG	3.6 ± 0.9^b^	10.8 ± 2.0^a^	<0.01	2.5 ± 0.7^b^	15.1 ± 4.5^a^	7.6 ± 2.3^ab^	5.3 ± 1.6^ab^	<0.01
PL	15.5 ± 0.9^b^	18.7 ± 0.8^a^	0.03	15.2 ± 1.4	18.0 ± 1.0	18.0 ± 1.6	17.0 ± 0.8	0.47
NEFA	1.7 ± 0.4	1.7 ± 0.3	0.88	1.2 ± 0.2^b^	2.2 ± 0.4^a^	1.7 ± 0.3^ab^	2.0 ± 0.4^a^	0.01
CE	1.1 ± 0.3	2.0 ± 0.4	0.09	0.9 ± 0.2^b^	1.7 ± 0.3^a^	1.9 ± 0.3^a^	1.6 ± 0.3^a^	<0.01
**Percent distribution**								
TAG	19.4 ± 4.0^b^	35.2 ± 3.7^a^	0.03	16.9 ± 2.5	42.1 ± 4.9	28.7 ± 7.5	22.0 ± 4.3	0.05
PL	69.0 ± 4.8^a^	55.5 ± 4.0^b^	0.05	79.4 ± 4.1^a^	48.8 ± 5.1^b^	56.4 ± 5.0^b^	63.2 ± 4.9^b^	<0.01
NEFA	7.5 ± 1.1	4.5 ± 0.7	0.05	5.3 ± 0.8	5.9 ± 0.9	5.3 ± 0.8	7.3 ± 0.9	0.17
CE	4.6 ± 0.8	5.4 ± 0.7	0.52	4.0 ± 0.7	4.4 ± 0.7	5.9 ± 0.8	5.8 ± 0.8	0.06

a,b *Within groups and sampling days resp., least square means with different superscript letters differ (P < 0.05)*.

1 *Lact2 = lactation 2, Lact≥3 = lactation 3 or greater*.

2 *a.p. = antepartum, p.p. = postpartum*.

**Table 2:** Least square means ± standard errors and F-test results (*P-*values) for non-esterified fatty acids (NEFA, μmol/l serum), ß-hydroxybutyrate (BHB; mmol/l plasma), and cholesterol (Chol, mmol/l plasma) in blood, and for the body condition score [BCS; according to Edmonson et al. (37)] of both groups and at four sampling days for both groups combined, and differences in body weight (BW, kg) between groups at four sampling days.

	**Group** ^**1**^			**Sampling day** ^**2**^				
	**Lact2**	**Lact≥3**	***P-*value**	**14d a.p**.	**7d p.p**.	**28d p.p**.	**42d p.p**.	***P-*value**
NEFA in blood	196 ± 29^b^	419 ± 49^a^	<0.01	176 ± 40^b^	698 ± 159^a^	268 ± 61^ab^	205 ± 47^b^	<0.01
BHB in blood	0.54 ± 0.09	0.51 ± 0.07	0.82	0.34 ± 0.07	0.56 ± 0.12	0.61 ± 0.13	0.65 ± 0.14	0.14
Chol in blood	3.96 ± 0.27	3.51 ± 0.21	0.24	2.59 ± 0.18^b^	2.57 ± 0.16^b^	4.65 ± 0.29^a^	5.12 ± 0.45^a^	0.02
BCS	2.55 ± 0.13	2.40 ± 0.10	0.37	2.97 ± 0.09^a^	2.30 ± 0.10^b^	2.36 ± 0.09^b^	2.30 ± 0.09^b^	<0.01
Δ BW				19.2	24.2	11.3	1.1	

a,b *Within groups and sampling days resp., least square means with different superscript letters differ (P < 0.05)*.

1 *Lact2 = lactation 2, Lact≥3 = lactation 3 or greater*.

2 *a.p. = antepartum, p.p. = postpartum*.

### Total Lipid Content of Liver Tissue

Cows of group Lact≥3 had a higher hepatic TL content than cows of group Lact2, and the concentration changed in the periparturient period in both groups (Table 1; Figure 1A). In cows of group Lact2, a numerical increase from d 14 a.p. to d 7 p.p. and a numerical decrease from d 7 p.p. to d 42 p.p. was observed. In cows of group Lact≥3, the TL content increased from d 14 a.p. to d 7 p.p. (*P* = *0.03*). Between d 7 p.p. and d 28 p.p. it was unaltered and decreased numerically to d 42 p.p. again. However, it was still significantly higher on d 42 p.p. (*P* = *0.02*) than on d 14 a.p. The concentrations tended to be higher on d 28 p.p. compared with d 14 a.p. (*P* = *0.07*). Comparison of the Lact≥3 and Lact2 groups on the same sampling day showed a significant difference on d 42 p.p. *(P* < *0.01)*, and on d 14 a.p. *(P* = *0.07)* and d 28 p.p. *(P* = *0.08)*, the groups tended to be different.

### Main Lipid Fractions

#### Hepatic TAG Content

Overall, Lact≥3 cows had significantly higher hepatic TAG contents than Lact2 cows, and the contents varied within the study period in both groups (Table 1; Figure 1B). In Lact2 cows, the TAG contents increased from d 14 a.p. to d 7 p.p. (*P* = *0.05*) and tended to decrease by d 42 p.p. (*P* = *0.05*). In Lact≥3 cows, the TAG contents increased from d 14 a.p. to d 7 p.p. (*P* = *0.01*) and d 28 p.p. (*P* = *0.01*), and on d 42 p.p. remained higher than on d 14 a.p. (*P* = *0.04*). Lact≥3 cows had significantly higher TAG contents on d 28 *(P* < *0.01)* and d 42 p.p. *(P* = *0.01)* compared with Lact2 cows on the same sampling days.

#### Hepatic PL Content

Overall, Lact≥3 cows had significantly higher hepatic PL contents than Lact2 cows, and the contents did not vary significantly within the study period (Table 1). Lact≥3 cows had significantly higher PL contents on d 14 a.p. (*P* = *0.04*) and d 42 p.p. (*P* = *0.02*) than Lact2 cows on the same sampling days.

#### Hepatic NEFA Content

The hepatic NEFA contents did not differ between groups but varied within the study period (Table 1). Independent of the lactation group, lower NEFA contents were observed at 14 d a.p. compared with 7 and 42 d p.p. In Lact≥3 cows, the contents tended to be higher on d 7 p.p. (*P* = *0.06*) and was significantly higher on d 42 p.p. (*P* = *0.02*) compared with d 14 a.p.

#### Hepatic CE Content

The overall CE contents tended to be higher in Lact≥3 cows than in Lact2 cows, and there were significant changes within the study period with a p.p. increase in both groups (Table 1; Figure 1C). In Lact2 cows, the CE contents increased from d 14 a.p. to d 7 p.p. (*P* = *0.01*) and at d 42 p.p. was higher than on d 14 a.p. (*P* = *0.03*). The CE contents tended to be higher on d 28 p.p. than on d 14 a.p. (*P* = *0.08*). In Lact≥3 cows, the CE contents increased from d 14 a.p. (*P* < *0.01*) and from d 7 p.p. (*P* = *0.04*) to d 28 p.p. The CE contents tended to decrease (*P* = *0.08*) after d 28 p.p. but tended to remain higher compared with d 14 a.p. (*P* = *0.07*).

#### Percentage Distribution of the Lipid Fractions in the TL Content

In cows of both groups, TAG and PL were the largest and NEFA and CE the smallest lipid fractions in TL (Table 1; Figure 2).

The percentages of TAG were significantly greater in group Lact≥3 than in group Lact2 (Table 1; Figure 2). The percentages of TAG tended to vary in the periparturient period. They were significantly higher on d 42 p.p. (*P* = *0.03*) and tended to be higher on d 28 p.p. (*P* = *0.06*) in group Lact≥3 compared with group Lact2 on the same sampling days.

The percentages of PL varied in the study period with higher levels a.p. than p.p. and were smaller in group Lact≥3 than in group Lact2 (Table 1; Figure 2). In group Lact2, there was a trend for a decrease from d 14 a.p. to d 7 p.p. (*P* = *0.06*) and a trend for a rebound by d 28 p.p. (*P* = *0.09*). In group Lact≥3, the percentages decreased from d 14 a.p. to d 7 p.p. (*P* = *0.01*), d 28 p.p. (*P* < *0.01*), and d 42 p.p (*P* < *0.01*). Cows of group Lact≥3 had significantly smaller PL percentages on d 28 (*P* = *0.01*) and d 42 p.p. (*P* = *0.04*) than cows of group Lact2 on the same sampling days.

The percentages of NEFA tended to be lower in cows of group Lact≥3 compared with group Lact2 and remained unaltered across the periparturient period (Table 1; Figure 2).

The percentages of CE did not differ between and within groups (Table 1; Figure 2).

### NEFA, BHB, and Cholesterol Concentrations in Blood

Overall, the Lact≥3 cows had significantly higher NEFA concentrations in blood than Lact2 cows, with a more than two times higher concentration, and there were significant changes in concentration over the periparturient period (Table 2; Figure 3A). The concentrations increased in both groups from d 14 a.p. to d 7 p.p. (Lact2: *P* = *0.02*; Lact≥3: *P* = *0.04*) and then decreased in Lact2 cows until d 42 p.p. (*P* = *0.04*). The NEFA concentrations on d 14 a.p. (*P* = *0.04*) and d 42 p.p. (*P* = *0.02*) were significantly higher in Lact≥3 cows compared with Lact2 cows on the same sampling days.

The BHB concentration was numerically higher after calving than before but did not differ significantly between the groups and among the sampling days (Table 2).

The cholesterol concentrations did not differ between groups but there were significant differences among sampling days (Table 2; Figure 3B). In Lact2 cows, the concentration increased from d 14 a.p. (*P* = *0.04*) and from d 7 p.p. (*P* = *0.02*) to d 28 p.p. and tended to be higher on d 42 p.p. than on d 7 p.p. (*P* = *0.09*). Overall, changes in the cholesterol concentration among sampling days were significant in Lact≥3 cows (*P* = *0.05*), and the concentrations on d 28 and d 42 p.p. were numerically higher than on d 14 a.p. (d 28 p.p., *P* = *0.08*; d 42 p.p., *P* = *0.08*) and on d 7 p.p. (d 28 p.p., *P* = *0.02*; d 42 p.p., *P* = *0.04*).

### BCS, BW, and Milk Yield

The BCS did not differ between groups but varied significantly among sampling days (Table 2; Figure 4A). In both groups, the BCS was significantly higher before calving compared with all three sampling days after calving. The loss in body condition was numerically larger in Lact≥3 cows than in Lact2 cows.

No significant differences were observed in BW (Table 2; Figure 4B). The mean BW was numerically greater in Lact≥3 cows than in Lact2 cows at all sampling days. The difference diminished during the study period and had almost disappeared by d 42 p.p.

**Figure 5:**
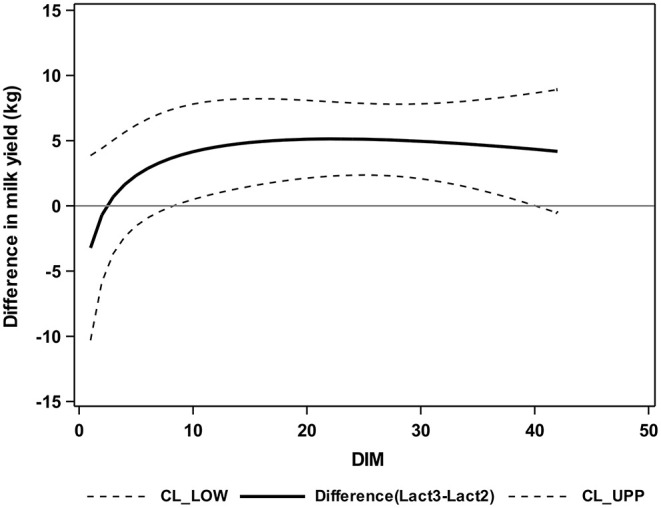
Differences between group Lact2 (lactation 2, *n* = 3) and group Lact≥3 (lactation 3 or greater, *n* = 5), least square means, and 95% confidence intervals for the milk yield (kg) of the two groups of German Holstein cows in the first 42 DIM. 0 = parturition; CL_LOW (lower dashed line) = lower confidence limit; CL_UPP (upper dashed line) = upper confidence limit; solid line = difference between groups.

Cows of group Lact≥3 had significantly higher milk yields from 9 to 40 DIM than Lact2 cows (Figure 5).

## Discussion

The main goal of this study was to generate primary data describing the dynamics of lipid fractions throughout the transition period in healthy dairy cows and to identify possible interrelations concerning the pathogenesis of FLS. The small sample size was a limiting factor in the interpretation and extrapolation of the results. However, the selected animals had similar and distinct changes in the level of production (BCS, milk yield), blood chemistry traits (NEFA, BHB), and liver lipid content as part of normal periparturient changes. Differences between the age groups (Lact2 and Lact≥3) were also seen and expected based on the results of other studies (4, 6, 7, 14, 18, 21, 33, 44–54). This supports the notion that the study cows were chosen carefully based on clinical findings and can be viewed as a homogenous cohort. Therefore, the results and interpretation of the dynamics of the lipid fractions have a valid basis and serve as a reference for future studies. Extrapolation of results on a large and heterogenous cohort that includes diseased animals is limited.

Our results corroborated previous observations that hepatic TL content increases in the periparturient period in dairy cows (6, 7, 48); the increase was significant in cows in lactation 3 or greater. This increase reflects mobilization of lipids from peripheral adipose tissue and hepatic uptake, metabolism, and storage of fat. Compared with younger cows, older cows mobilized considerably larger amounts of fat, particularly after calving, in the form of NEFA, which were then deposited in the liver. This confirmed earlier findings (4, 6, 7). The increase in hepatic TL content was merely numerical in second-lactation cows, and at the end of the study period, the hepatic TL content remained increased in older cows and was significantly higher than in younger cows. This shows that lipomobilization in older cows is not only more pronounced but also longer lasting than in younger cows. Likewise, serum NEFA concentrations were considerably higher in older cows as early as 2 weeks before calving and remained higher until the end of the study. Increased NEFA concentrations usually precede fat deposition in the liver (4, 21, 45). Lipomobilization was also reflected by the BCS (44), which decreased significantly in both groups. Of note, the mean weight of the older cows was numerically greater than that of the younger cows, but the difference disappeared by the end of the study, which supports the observation that the older cows mobilized considerably more body tissue. The most likely reason for this is the greater milk yield in older cows. Multiparous cows produce more milk than primiparous cows (33), and one study showed a positive association between milk yield in early lactation and hepatic TAG content (45). In accordance with the higher degree of lipomobilization, resolution of fatty liver takes longer in older cows than in younger cows and was not completed by the end of the study period. In previous studies, the hepatic TL content had returned to pre-calving levels by 8 weeks p.p. (14) and 65 DIM (48). Fatty liver is generally considered a reversible process (14, 48, 49), and up to 50% of all postpartum dairy cows have moderate to severe TAG accumulation in the liver (45). Cows can generally cope with some degree of hepatic TAG accumulation, which is a regular occurrence in early lactation and can be mitigated, albeit not prevented, through transition cow feeding and management strategies (55, 56). When cows are not able to adjust to metabolic changes in early lactation, ketosis and severe FLS may result (57). However, this did not happen in the present study; all of the eight cows remained clinically healthy, and the BHB concentration remained below the threshold of subclinical ketosis [1.2 mmol/l; (46, 47)].

In both groups and at all sampling days, TAG and PL formed the largest lipid fractions, which was in agreement with earlier findings (14). The hepatic TAG content increased markedly after calving and was responsible for most of the hepatic fat deposits following lipomobilization (21, 49). Periparturient increase in TL is largely based on increase in TAG content, and thus hepatic TAG concentration is considered representative of total hepatic fat content (14, 50, 51). Our findings showed that periparturient lipomobilization, which was evidenced by increased blood NEFA concentrations and loss of body condition and weight, caused an increase in the hepatic TAG concentration. This phenomenon was more pronounced in older cows, which had greater hepatic TAG contents, and confirmed earlier findings (6). However, the pattern of hepatic TAG content increase differed between the two groups. Older cows had a progressive increase from before calving to d 28 p.p., after which the concentration decreased slightly but remained above the prepartum level. In younger cows, the maximum was reached on d 7 p.p., and the concentration on d 42 did not differ from that on d 14 a.p. Other authors reported increased hepatic TAG content 28 d p.p. in dairy cows with a mean age of 6.0 years (18). The more severe and longer lasting lipomobilization and hepatic fat deposition seen in older cows is also reflected by the profile of the TAG content, increased blood NEFA concentrations, and decreasing BW. The most likely explanation for this is the greater milk yield in older cows.

Hepatic PL content did not differ between sampling days, which was in agreement with an older study (18). The main function of PL is the assembly of cell membranes (58). A fatty liver is larger than a normal liver (59), but the enhanced volume is likely not caused by an increase in the number of liver cells and is therefore not associated with an increase in cell membranes. However, older cows had higher hepatic PL content than younger cows, which could be related to higher hepatic TAG (and CE) concentrations. As TAG and CE are stored as lipid droplets in the cytoplasm of liver cells, these droplets are coated with a monolayer of PL (60) and the degree of fat deposition in the liver correlates positively with the size of lipid droplets (34). Consequently, knowing that older cows have higher hepatic TAG (and CE) content than younger cows, they can be assumed to have either larger or more lipid droplets, or both, and thus more PL per gram liver FW. The export of TAG as VLDL (61, 62) could also be related to an increased demand of PL, particularly phosphatidylcholines, in older cows with higher hepatic TAG content. Interestingly, the percentage of PL was higher in the younger cows. Other authors reported that the percentage of PL decreased with increasing TL content and percentage of TAG in TL in early lactation (14, 52). The percentage of PL describes the amount relative to TL and not the amount relative to liver FW that is reported for the hepatic PL content. The hepatic PL content did not change during the study period. However, liver cells containing large lipid droplets increase in volume (63), which in older cows could result in a decrease in the percentage of PL in TL through displacement by TAG because the latter increased in relative terms and thus consequently in volume. This is supported by the observation that the percentage of PL in TL in younger cows was decreased only on d 7 p.p. in contrast to at all three sampling days p.p. in older cows. At the same time, the hepatic TAG content was highest in younger cows on d 7 p.p., and significantly higher in older cows on d 28 and d 42 p.p. At these two sampling days, the percentage of hepatic PL was significantly lower in older cows compared with younger cows. Thus, it seems plausible that the percentage of hepatic PL was decreased in the older cows because of displacement by the massively increasing amounts of TAG.

Increased blood levels of NEFA reflect lipomobilization. The hepatic NEFA content did not differ between the groups, but older cows had higher blood concentrations. This speaks to the ability of the liver to metabolize considerable amounts of NEFA (64, 65) through synthesis of TAG. Furthermore, the ability of the liver to compensate is also reflected by the blood BHB concentrations, which increased numerically after calving but did not differ significantly between the groups and sampling days. The higher hepatic NEFA content in older cows on d 7 and d 42 p.p compared with pre-calving levels (52) could be related to their function as an energy substrate when demand is high. The percentage of NEFA did not change during the periparturient period (14) but tended to be lower in older cows. Comparison of the percentages of TAG, which increased considerably in early lactation, and those of NEFA, which increased only slightly, suggests that TAG displaces NEFA in liver tissue analogous to PL.

The hepatic CE content increased after calving in agreement with a previous report of increasing CE concentrations associated with increasing hepatic TL content (52). This association could explain why older cows, which had a higher hepatic TL content than younger cows, tended to have a higher CE concentration. The similarity between the periparturient concentration patterns of CE and TAG also suggest an association between the two lipid fractions. Older cows had the highest CE (and TAG) content on d 28 p.p. and younger cows on d 7 p.p. Thereafter, the CE content in younger cows did not decrease to the same extent as TAG, even though on d 28 p.p. it was numerically lower than in older cows, and on d 42 p.p it was still higher than before calving. The increased CE concentrations in early lactation indicate that the liver reacts to the increased demand for cholesterol (17) for synthesis of VLDL, which is required for export of TAG from the liver (62). However, it appears that the availabilities of cholesterol and CE are limited in older cows because TAG continues to be accumulated in the liver. The accumulation of TAG in the liver is negatively correlated with apolipoprotein B levels (66), and this affects the synthesis and secretion of VLDL and the uptake of CE by the liver (62) and further impairs the export of TAG. The plasma cholesterol concentration increased in early lactation as reported previously (17, 53, 54) but it is not clear why the concentrations were low before and 1 week after calving. Possible reasons included reduced feed intake associated with the expanding uterus, increased steroid hormone synthesis near the time of parturition, and secretion of cholesterol in the colostrum and milk (17, 54). In accordance, Newman et al. (24) observed no decrease in serum cholesterol in the periparturient period in cows on a ration high in metabolizing energy (adequate, not excessive), in contrast to cows receiving a low plane of nutrition.

Determination of the hepatic TL content and its lipid fractions requires liver biopsy samples and biochemical analytical methods. The concentration of TAG can be measured by means of biochemical or histologic analysis of liver tissue samples (50) but it is also possible to estimate the hepatic TAG content using a computer-aided ultrasound technique (67). Ultrasonographic or histologic measurement of other lipid fractions has not been described. Histologic measurement of hepatic TL content is problematic and only feasible when the hepatic TAG content is high. Histologic estimation of the TL content is based on the measurement of TAG, and when the TAG content is low, it is masked by the basal levels of other lipid fractions (50). Therefore, liver biopsy was a critical requirement based on the goals of this study even though it is an invasive procedure. Our findings were in agreement with the results of other studies on hepatic TL (48, 52), TAG (21, 49, 52), PL (21, 52), NEFA (14, 52), and CE (53) concentrations. Therefore, the methodology used in the present study was appropriate for the investigation of periparturient changes in the hepatic TL content and in the absolute and relative concentrations of lipid fractions, and for comparison of cows of two age groups with respect to these variables.

## Conclusions

The findings of this study confirm lipomobilization, as a result of the onset of lactation, and associated fat deposition in the liver of dairy cows. Increasing milk yield in early lactation accounts for the increased energy demand and the decrease in BCS, and the increasing NEFA concentrations in blood reflect the reaction of the cow to the energy deficit. The higher milk yield in older cows may have resulted in mobilization of more peripheral fat than in younger cows and therefore deposition of more TL and TAG in the liver. Minimally increased BHB concentration in blood testifies to the ability of the liver to compensate for transient energy shortages.

Both the absolute and relative hepatic concentrations of the lipid fractions varied considerably in the periparturient period. TAG is the main lipid fraction deposited and stored in the liver and its content in liver tissue is higher in older cows. The hepatic content of structural lipids (PL) varied little in the periparturient period, but older cows had higher levels. This could be related to the storage of TAG and CE in lipid droplets or to the increased demand for PL for VLDL synthesis, or both. The observation that the hepatic NEFA content did not differ between older and younger cows despite considerably higher blood NEFA concentrations in the former speaks to the non-age-related ability of the liver of the dairy cow to oxidize and re-esterify NEFA.

The present study confirmed that lipomobilization and hepatic fat storage in dairy cows are reversible processes that do not necessarily cause clinical disease. Furthermore, our hypothesis that the hepatic TL content and the absolute amounts of lipid fractions, as well as their relative amounts, accumulated in the liver, differ between younger and older dairy cows in early lactation, was accepted. This could explain the increased risk of older cows for developing metabolic, infectious, and reproductive disorders associated with FLS. This should be investigated in future studies with a greater sample size and a more elaborate study design involving different age groups and health statuses.

## Data Availability Statement

The raw data supporting the conclusions of this article will be made available by the authors, without undue reservation.

## Ethics Statement

The animal study was reviewed and approved by the experiment was conducted in accordance with the German Animal Welfare Act and the Saxony Veterinary and Food Safety Office Landesdirektion Sachsen, permit number TVV 33/15. Written informed consent was obtained from the owners for the participation of their animals in this study.

## Author Contributions

KT and AS: conceptualization. KT, TS, FP, ST, AL, and MS-B: data curation. JS, SP, and EB: formal analysis. AS: funding acquisition and supervision. KT, TS, FP, ST, A-SL, MS-B, AS, KR, GD, and HF: investigation. JS, SP, and EB: methodology. AS, KT, TS, FP, A-SL, and ST: project administration. AS and HF: resources. AS and KT: validation. KT, JS, and SP: visualization. KT: writing – original draft. AS, WB, MS-B, JS, KT, KR, GD, and HF: writing – review & editing. All authors contributed to the article and approved the submitted version. Classified according to the CASRAI CRediT – Contributor Roles Taxonomy (https://casrai.org/credit/).

## Funding

This work was supported by Bayer Animal Health GmbH with a stipend and project support and by ESAOTE Biomedica Deutschland GmbH with financial support. We acknowledge support from Leipzig University for Open Access Publishing.

## Conflict of Interest

The authors declare that the research was conducted in the absence of any commercial or financial relationships that could be construed as a potential conflict of interest.

## Publisher's Note

All claims expressed in this article are solely those of the authors and do not necessarily represent those of their affiliated organizations, or those of the publisher, the editors and the reviewers. Any product that may be evaluated in this article, or claim that may be made by its manufacturer, is not guaranteed or endorsed by the publisher.

## Abbreviations

a.p.: antepartum
BCS: body condition score
BHB: ß-hydroxybutyrate
CE: cholesterol esters
DIM: days in milk
FLS: fatty liver syndrome
FW: fresh weight
Lact2: lactation 2
Lact≥3: lactation 3 or greater
NEFA: non-esterified fatty acids
p.p.: postpartum
PL: phospholipids
TAG: triacylglycerol
TL: total lipid
VLDL: very-low-density lipoprotein.
